# Impaired cognitive functioning in stress-induced exhaustion disorder: a new tablet-based assessment

**DOI:** 10.1186/s12888-021-03454-1

**Published:** 2021-09-18

**Authors:** Aniko Bartfai, Marie Åsberg, Aniella Beser, Kimmo Sorjonen, Alexander Wilczek, Siegbert Warkentin

**Affiliations:** 1 Department of Clinical Sciences, Karolinska Institutet, Danderyd University Hospital, 182 88 Stockholm, Sweden; 2 Department of Clinical Neuroscience, Division of Psychology, Karolinska Institutet, 171 77 Stockholm, Sweden; 3 Department of Psychology, Faculty of Health and Life Sciences, Linnaeus University, 351 95 Växjö, Sweden

**Keywords:** Stress- induced exhaustion disorder, Cognitive impairment, Attention lapses, Tablet-based neuropsychological tests

## Abstract

**Background:**

The adverse health effects of stress induced exhaustion disorder (SED) cause increasing concern in Western societies. This disorder is characterized by severe fatigue, decreased tolerance to further stress, and attention and memory lapses. Despite subjective complaints, individual cognitive deficits are not always detected in a clinical setting, which calls for the validation of more sensitive instruments.

**Aim:**

The objective of this study was to investigate if a short, tablet-based serial naming task, MapCog Spectra (MCS) could be used as a marker for cognitive problems in SED.

**Participants:**

The study comprised of 39 subjects (35 females, four males) with SED. Their mean age was 46,8 years (SD 10.1; range 30–60 yrs.). All participants were healthcare professionals, with a college or university degree, doctors, registered nurses, and psychologists.

**Methods:**

The MCS was used to assess the number of aberrant pauses during serial naming of coloured geometrical shapes. The Coding, Matrix Reasoning, Digit Span, Symbol Search of the WAIS-IV, and RUFF 2&7 tests, were administered together with a short interview.

**Results:**

Mean values were within normal reference limits for all tests, except for the MCS, which showed a significantly higher number of aberrant pauses (*p* < 0,001) in the SED group, compared to normal reference values. Although subjects performed within normal limits on the RUFF 2&7, a significant difference between individuals was found in the performance strategy of the participants.

**Conclusion:**

Here we report that subjects with SED have performance deficits on the MCS, in terms of aberrant pause times, despite average performance on WAIS-IV tests measuring inductive reasoning, processing speed, working memory, and attention. We also demonstrate that subjects use different strategies to overcome their problems. These findings add to the growing evidence of cognitive deficits in SED and that the MCS might aid neuropsychologists in disentangling cognitive markers, important to substantiate the subjective complaints of affected individuals.

## Introduction

The adverse health effects of occupational stress have caused increasing concern in most Western countries [1]. The association between occupational stress and coronary events has been established beyond reasonable doubt [2]. The potential hazards on mental health are less well known, but there is evidence that prolonged stress may result in a state of exhaustion, characterized by symptoms reminiscent of minor brain dysfunction, i.e., a syndrome of severe fatigue, cognitive problems, and decreased tolerance to further stress [3].

This syndrome was recognized already in the late nineteenth century and called acquired neurasthenia [4]. As the term neurasthenia fell into disuse in the early twentieth century [5] the syndrome received various other diagnostic labels. It has sometimes been seen as a variety of depressive illness, exhaustion depression [6] or job stress depression [7], as well as the final stage of a professional burnout process (“severe” or “clinical” burnout). The lack of consensus about the diagnosis, the alleged vagueness of the symptomatology and absence of objective findings may have caused problems for the patients, who sometimes feel distrusted by their physicians as well as by the health insurance system.

In 2005, the Swedish Board of Health and Social Welfare recommended the term Stress-related Exhaustion Disorder (SED; 43.8A in the ICD-10) for the condition, and tentative diagnostic criteria were formulated [8]. In international literature this condition is often referred to as Burnout or Clinical burnout. Burnout is a syndrome, but not a medical condition [9–12], which is characterized by fatigue, reduced professional efficacy and cynicism rather than the cognitive problems characteristic of SED.

Cognitive symptoms are among the most prevalent and pervasive complaints in SED. Patients complain of concentration and memory lapses in everyday tasks, slowed thinking and reduced mental capacity for sustained effort or flexibility [10–12]. The approach to measure cognitive complaints is not standardized and varies between studies. Cognitive complaints in SED can be measured subjectively by questionnaires [13, 14] or objectively by neuropsychological tests [13–18]. Sandström and collegaues. Found, for example, that patients performed significantly poorer on tests for immediate and delayed recall of non-verbal memory, as well as auditory and visual attention, but they performed equally well on measures of general cognitive ability and verbal memory compared to controls. Subsequently, Sandström and co-workers [17, 18] found that patients performed significantly poorer on measures of attention and response control, as well as visuo-spatial memory ability. Jonsdottir and coworkers [19] reported that patients performed significantly poorer on tasks requiring speed, attention span, cognitive control, learning, working memory and episodic memory, compared to controls. Rydmark and coworkers [16], found that female patients performed less well than controls on tests of working memory and reaction time.

On the other hand, Öhman and coworkers [14] found no group differences in non-verbal memory, auditory and visual attention, but observed inferior performance for patients on tests of letter fluency, for Trail Making Test, Digit Symbol and for tests of prospective memory. The authors suggest that these findings reflect deficits in executive control and the functioning of the prefrontal cortex. Oosterholt [15] examined clinical and nonclinical burnout groups, and found that both reported cognitive problems, but evidence for impaired cognitive test performance was only found in the clinical burnout group, showing impaired general cognitive processing speed deficits, but no impairment in executive functioning.

There are apparent disparities in results between different studies, but the general overall picture of cognitive dysfunction in SED [11, 12] points toward a cognitive dysfunction subserved by the frontal executive and attention systems. One of the possible reasons for differences in results in the literature is probably methodology i.e., differences in the selected clinical neuropsychological tests. Clinical neuropsychological tests are complex and multi-faceted by the very nature of the task. Varying conditions, different modes of presentation or response requirements, etc. might trigger or attenuate a cognitive impairment. We believe that the earlier results concerning cognitive impairment might be best reconciled with impaired function in the executive attention system [20, 21]. This interpretation is supported by neurophysiological [22] and brain imaging studies of SED [23, 24], which have shown structural and functional changes in prefrontal areas [12, 17] in the anterior cingulum and limbic regions [25], i.e., brain areas which have previously been identified as subserving executive attention.

Cognitive functions, such as speed of processing, attentionspan, or working memory and episodic memory are rarely discernible as discrete entities, but they are rather formed by the intrinsic cooperation of many subsystems, which might also be utilized by other cognitive functions. Attention is one example of such a ubiquitous function of the brain that plays a critical role in most cognitive abilities and everyday functioning. For the SED syndrome there is a need to develop an easily administered cognitive test indicator serving as a cognitive marker which also could be used during follow-up, since the cognitive symptoms might prevail for years.

These considerations led us to explore the potential of a newly developed, tablet-based test, the MapCog Spectra (MCS) for SED patients. This new tablet-based test has its conceptual roots in the rapid automatized naming (RAN) tradition [26]. In this tradition subjects are requested to name arrays of 50 stimuli (i.e., letters, digits, shapes or objects) as quickly as they can, and the outcome measure is the total naming time. The MCS-test features instead a combination of two stimuli dimensions (i.e., colour and shape combinations) which we believe adds a between-domain and within-domain attentional switch-cost to the test performance [27]. Our preliminary findings with this approach confirm previous RAN-findings, in that the moment-to-moment pause times during the naming procedure are longer and more variable in such diverse populations as Alzheimer’s disease and neurodevelopmental disorders [28, 29]. The pause times were not significantly correlated with reaction time [30] or premorbid intelligence.

Thus, the aim of the present study was to explore the diagnostic potential of the MapCog Spectra and validate the results through simultaneously obtained data on clinical neuropsychological tests, which in previous studies have indicated sensitivity to cognitive impairment in SED patients [16–19].

## Methods

### Design and setting

The study was conducted at the Department of Psychiatry of the Ersta Hospital psychiatric clinic in Stockholm, Sweden. The clinic specialized in treating health care professionals, and all patients were working in various healthcare units in the larger Stockholm catchment area. Patients were referred by their general practitioners or were self-referred. Typically, a diagnosis was formulated after three sessions with a senior psychiatrist, an M.D. together with a psychiatric nurse. Patients diagnosed with SED received written information about the present study and were invited to participate. Due to unforeseen external factors, data collection had to be terminated earlier than planned, limiting the number of participants.

### Study population

In total, 49 subjects with SED as the primary diagnosis participated in the study. After controlling anamnestic data, nine subjects with a history of cerebral trauma or illness, a history of psychosis, bipolar disorder, or substance abuse were excluded. One additional subject was excluded due to a technical error in the registered data. Thus, the study population comprised of 39 subjects (35 females, four males). The mean age was 46,8 years (SD 10.1; range 30–60 yrs.). All participants were healthcare professionals, with a college or university degree, doctors, registered nurses, and psychologists. Diagnostic criteria are presented in Table 1.

**Table 1:** Criteria for Exhaustion Disorder according to the Swedish National Board of Health and Welfare^a^

A. Physical and mental symptoms of exhaustion during at last two weeks. The symptoms have developed in response to one or more identifiable stressors present for at least six months.	
B. The clinical picture is dominated by markedly reduced mental energy, as manifested by reduced initiative, lack of endurance, or increased time needed for recovery after mental effort.	
C. At least four of the following symptoms have been present, nearly every day, during the same 2-week period:	
1. Concentration difficulties or impaired memory	
2. Markedly reduced capacity to tolerate demands or to work under time pressure	
3. Emotional instability or irritability	
4. Sleep disturbance	
5. Marked fatigability or physical weakness	
6. Physical symptoms such as aches and pains, palpitations, gastrointestinal problems, vertigo, or increased sensitivity to sound	
D. The symptoms cause clinically significant distress or impairment in occupational, social or other important respects	
E. The symptoms are not due to the direct physiological effects of a substance (e.g. a drug abuse, a medication) or a physical illness/injury (e.g. hypothyroidism, diabetes, infectious disease).	

^a^ Criteria A-E must be fulfilled to diagnose SED

Regarding treatment with psychotropic drugs, specific data for the included subjects were lacking. However, they were all participating in the clinic’s SED program. In this program, 33% of the subjects (*n* = 153) were treated with SSRI, 11% with SNRI, 29% with anxiolytics, and 33% were treated with hypnotics.

### Procedure

Before starting the neuropsychological assessment, the participants were asked to rate their symptoms on a VAS scale, ranging from 1 -to -10, of their a) current level of exhaustion b) perceived level of exhaustion at its highest level.

A psychologist provided detailed information about the test session. The session started with a short anamnestic interview regarding possible earlier brain injury or disease. The clinical neuropsychological tests were administered in the following fixed order: Coding, MapCog Spectra, Ruff 2&7, Symbol search, Digit Span, and Matrix Reasoning (see description below). They were all conducted in one session with an average duration of 75 min.

### Measurements

**Fig. 1:**
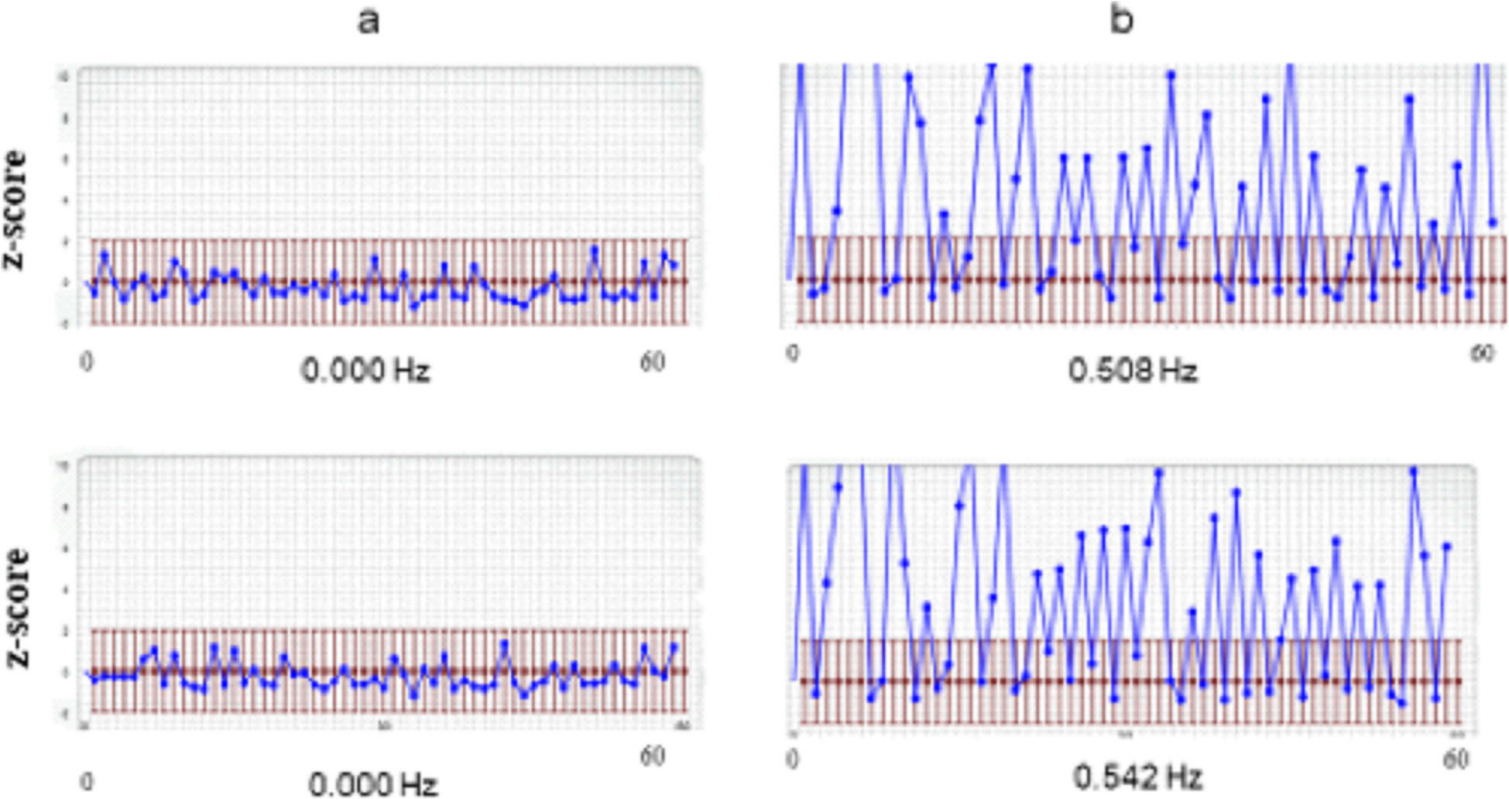
Response patterns from a normo-typical person (**a**) and an adult with ADHD (**b**). The graph shows 60 consecutive pause times (horizontal X-axis) and their duration transformed to z-scores (vertical y-axis). The red bars denote +/− 2 standard deviations (SD) of z-scores. Note that in Figure 1**a** all pause times of this individual (blue dots) lie below 2 SD’s of normo-typical performance. The performance is denoted below the curve as 0.000% (i.e., number of aberrant pauses above 2 SD is 0/60 = 0.000%). **b** displays a typical response pattern from an adult with ADHD. Note that many z-scores exceed 2 SD in both trials, denoting a high frequency of aberrant pause times

The MapCog Spectra (MCS) standardized measurement procedure was performed on a tablet (iPad), and commenced as follows: First, a short training session was conducted wherein the subject was asked to name four different colour and shape combinations (e.g., red heart, black ball, blue cube, and yellow star) displayed on the tablet. As the methodology is based on sound recordings detecting pause time durations between verbal responses, the subjects were instructed to avoid extraneous sounds (e.g., coughing, laughing, etc.). After a successful training session, a grid of 40 (5 × 8) randomly ordered colour-shape combinations appeared on the screen (i.e., the same colours and shapes as in the training session, but re-randomized in different combinations). The subjects were asked to name the stimulus combinations beginning from top left to the bottom right of the screen and were also instructed to name the colour first and then the shape. After each one of the 40 combinations was named, a second trial was performed. The time between the two trials was less than 30 s. The stimulus combinations were automatically re-randomized between the two trials to avoid learning effects. After completing the second trial, the results automatically appeared on the screen in a graph (Fig. 1).

The graph in Fig. 1 shows 60 consecutive pause times (horizontal X-axis), and their duration transformed to z-scores (vertical y-axis). The red bars denote +/− 2 standard deviations (S.D.) of z-scores. Note that all pause times of this individual (blue dots) lie below 2 SD of normo-typical performance. The performance is denoted below the curve as 0.000% (i.e., the number of aberrant pauses above 2 SD is 0/60 = 0.000%).

Only the occurrence of pauses longer than 100 milliseconds was recorded due to an increasing inaccuracy of identifying the borders of sound onset and offset of pause time durations shorter than this time limit. The identification of the boundaries between speech offset and onset was automatically performed by Fast- Fourier analysis of the wave-files.

After 60 successive pauses had been recorded, the identification of additional pauses was automatically stopped.

Thereafter, the duration of each individual pause time was automatically recalculated into z-values. This recalculation was based on the total number of typical reference values included in the database of the test (*n* = 347, ages 10–87 years). This database of reference values comprises normo-typical school children (*n* = 164), university students (*n* = 53), and healthy elderly who had responded to local advertisements (*n* = 130). Subgroups of these reference values are included in several publications [28, 31, 32].

The reason for the recalculation into standardized z-values was that the means of the successive pause time durations differed between each other, as did the standard deviations. Based on this recalculation, 60 z-values were plotted on the resulting graphical presentation of pauses obtained during a subject’s performance. In addition, the graphical presentation also includes an upper limit of 2 standard deviations for each z-value. An aberrant pause time means that the pause time duration lies above 2 standard deviations from the reference value. Each pause time is also depicted in a scroll list, which shows at which time the registered pause occurred, the corresponding z-value, and if the pause was aberrant or not.

The number of aberrant pauses of a performance was defined as follows: During a normal performance, an upper limit of 6 aberrant pauses was allowed. This was motivated with reference to Sonuga-Barke and Castellanos [33] who hypothesized that the brain’s default mode network could interfere with test performance with a frequency of 0.01 < Hz < 0.1. Therefore, a total of 6 aberrant pauses (of the total number of pauses) were included in a normal performance.

### Clinical neuropsychological tests

The selection of clinical neuropsychological tests, containing predominantly processing speed and attention, was based on results from earlier studies [13–15, 17] which used more extensive neuropsychological test batteries and consequently found impairment in these cognitive areas. Due to the core symptom of exhaustion, we tried to shorten the testing period. Matrix Reasoning was included as an estimate of premorbid IQ.

### Tests from the Wechsler adult intelligence scale-IV [34, 35]

Digit Span (DS*).* The task is a measure of working memory capacity, i.e., the cognitive ability to store and manipulate information transiently.

Matrix Reasoning (M) is a test of nonverbal abstract problem solving and inductive reasoning. The task is to view a series of increasingly difficult visual patterns and select the picture that fits the array from five options. The test was used as a test of inclusion to ensure an adequate level of reasoning skills and abstract thinking for the remaining tasks.

Symbol search (S.S.) The task is to determine whether a target symbol appears among various simple symbols. The subject is required to mark either the yes or no checkbox with a pencil in response to as many items as possible within 2 min. The test is designed to assess information processing speed and visual perception.

Coding is a pencil and paper test of psychomotor performance in which the subject is given a key grid of numbers and matching symbols and a test section with numbers and empty boxes. The test consists of filling as many empty boxes as possible with a symbol matching each number. The test demands attentional control, attentional switching, graphomotor control, associative learning, visuospatial processing, and executive control/cognitive control.

Test scores are presented both as raw scores and as age corrected scaled scores according to the manual.

### The Ruff 2 & 7 selective attention test

*This test was developed to assess sustained and selective visual attention* [36]*.* The test consists of a series of 20 trials of a visual search and cancellation task. Each trial lasts 15 s (in total 5 min). The respondent detects and marks through all occurrences of the two target digits: 2 and 7. Two types of trials are presented in the test: Automatic detection trials, in which target numbers are presented among distractor letters, and Controlled Search trials, in which target numbers are presented among distractor numbers. Selecting targets from different stimulus categories represent parallel search or automatic information processing. In contrast, selecting targets from the same stimulus category requires a serial search or controlled information processing. Thus, working memory and effortful processing of stimulus characteristics are needed to select targets from distractors effectively. The following scores are used: Automatic Detection Speed, the raw score representing the sum of correct letter trials; Automatic Detection Accuracy (ADA), where the number of errors modifies the raw ADS score; Controlled Search Speed (CSS), i.e., the total number of hits in the number trials; Controlled Search Accuracy (CSA), where the number of errors modifies the raw CSS score. According to the T-scores manual, raw scores were corrected for age and educational level [37].

Further, composite scores were created for Total Speed and Total Accuracy by summing the T- scores for ADS and CSS, and ADA and CSA respectively. Finally, a Discrepancy Analysis was conducted based on the differences between the two speed and the two accuracy T-score. The absolute value of this difference constitutes the Total Difference score with higher values indicating increasing difficulties in coping with issues of selective attention. The manual provides significance levels associated with Speed, Accuracy, and Total Difference scores, indicating impaired performance compared to the normative group. Test scores are presented both as raw scores and as corrected scaled scores for age and educational level according to the manual.

### Statistical analyses

The means and standard deviations of the raw scores of each test were used in the analyses. Regression analyses and correlation analyses (Pearson’s correlations) were used to compare the mean number of aberrant pauses of the two MCS trials (dependent variable) and the WAIS subtests and the RUFF 2&7 (independent variables). A stepwise regression analysis was also performed to assess which predictor variables accounted independently for MCS variance. Sensitivity, specificity, the ROC curve, and Area Under the Curve (AUC) when using MCS to discriminate between SED patients and healthy controls was calculated employing R-4.0.2 statistical software [38] and the pROC package [39]. The Youden index [40] (= sensitivity + specificity - 1) was defined for each point on the ROC curve and the point giving the largest index value was selected as a cut-off. Power, i.e., the probability to receive a significance level = 0.05, was calculated for the significant findings assuming that the population effect size equals observed sample effect size.

### Ethics

Patients were informed about the study by a written letter. Potential participants were provided with detailed information by the research psychologist before they signed informed consent. All participants could withdraw their consent to participate at any time. The Regional Ethical Board in Stockholm, Sweden approved the study (Dnr: 2017/2298–31/2).

## Results

No test scores were significantly related to age. Due to the minimal number of men among the participants, possible gender differences in performance could not be calculated. Data collection was stratified into four subject groups depending on duration since diagnosis, to control if the time of SED affected scores. The first subject group had an illness duration of 0–3 months (*n* = 7), the second group 4–6 months (*n* = 12), the third group 7–9 months (*n* = 5), and the fourth group 10–12 months (*n* = 15) following diagnosis. There were no significant differences regarding MCS-results between these groups. Thus, MCS data were calculated for the whole group only. As rated by the VAS scale, the mean level of current exhaustion was 4,82 (range 1–9), and the mean of the highest level of exhaustion was 8,74 (range 7–10).

The mean frequency of aberrant pause times for the two MCS-trials was 15.9% (SD 9.1%, range 0.00–33.4%), similar to other patient populations [41]. The number of aberrant pauses (above 10,0%) in the SED group was 29/39 (74.4%), while the corresponding number was 2/64 (3,1%) in the reference group.

The mean values for the neuropsychological tests from WAIS-IV are presented in Table 2 and for the Ruff 2&7 test in Table 3.

**Table 2:** AIS-IV, mean, range and scaled scores

WAIS-IV - subtests	Mean score (SD)	Range	Mean scaled Score (SD)
Matrix Reasoning	17.3 (5.0)	6.0–32.0	9.6 (3.4)
Digit Span	24.4 (4.5)	17.0–39.0	9.8 (2.3)
Symbol search	33.2 (6.8)	19.0–47.0	11.3 (2.7)
Coding	62.7 (12.9)	35.0–88.0	10.1 (2.5)

Age-corrected standard scores for each WAIS-IV test indicate the subjects’ performance was within the normal range

**Table 3:** Mean raw scores and mean T scores for the Ruff 2&7 test

RUFF measures	Mean raw score (SD)	Mean T-score (SD)
Ruff 2&7 test		
ADS^a)^	135.77 (28.27)	46.92 (9.15)
ADA^b)^	96.94 (3.21)	49.87 (7.78)
CSS^c)^	119.72 (24.16)	45.38 (9.85)
CSA^d)^	94.77 (4.45)	51.54 (8.80)

^a^ADS denotes Automatic Detection Speed

^b^ADA represents Automatic Detection Accuracy

^c^CSS indicates Controlled Search Speed

^d^CSA denotes Controlled Search Accuracy

For the Ruff 2&7 test, performance for sustained and selective attention was within the normal range, as shown in Table 3. Further analyses of the differences between automatic (letter and digit stimuli) and effortful (only digit stimuli) processing for total speed, total accuracy, and total scores indicated an irregular pattern for the results (Table 4). The results for total speed showed an unusually large discrepancy. About two-thirds of the participants (62%) exhibited a significant (*p* < .01), difference between automatic detection and controlled search compared to 2% of the participants in the normative group (effect size (w) = 4,29, *p* < 0.001).

**Table 4:** Discrepancy analysis of the Ruff 2&7 test. Number of subjects with differences between automatic (ADS) and effortful stimulus conditions (CSS), i.e., Total Speed Score and Total Accuracy Score and for the Total Score

Levels of significance	Discrepancy analysis		
	Total speed^**a**^	Total accuracy^**b**^	Total difference
**ns**	23	26	12
***p*** **< .10**	2	2	1
***p*** **< .05**	5	2	2
***p*** **< .01**	9	9	24
**Total nr**	39	39	39

^a^Total speed = **|**ADS-CDS**|**

^b^Total accuracy = **|**ADA–CSA**|**

Thus, the results indicate that a high proportion of SED subjects exhibit difficulties with selective attention defined as an ability to switch between automatic detection and controlled search despite results within the normal range for both speed and accuracy, i.e., many subjects managed to compensate for this impairment by adjusting performance by a strategy favouring either speed or accuracy in performance.

Correlations between MCS and the other neuropsychological tests are presented in Table 5. Strong correlations were obtained between MCS and tests reflecting processing speed and attention (Coding, Symbol Search, and Ruff 2&7 speed). Matrix Reasoning and Digit Span did not correlate significantly with MCS, but Digit Span was significantly correlated with Symbol Search and Ruff 2&7 CSS.

**Table 5:** Correlation coefficients for the cognitive tests (*n* = 37). MCS, is expressed in mean %. Matrix Reasoning, Digit span, Coding, Symbol Search (WAIS-IV), and Ruff 2&7 are expressed in mean raw scores

	2	3	4	5	6	7	8	9
1. MCS	−0.303	−0.033	− 0.504**	−0.644****	− 0.512**	−0.109	− 0.482**	−0.055
2. Digit.span		0.105	0.401*	0.28	0.347*	0.159	0.4*	− 0.098
3. Matrix Reasoning			0.093	0.122	0.296	0.178	0.356*	0.167
4. Symbol.Search				0.683****	0.505**	0.066	0.495**	−0.011
5. Coding					0.501**	0.052	0.533**	0.109
6. Ruff 2&7 ADS						0.022	0.816****	−0.37*
7. Ruff 2&7 ADA							−0.122	0.434**
8. Ruff 2&7 CSS								−0.215
9. Ruff 2&7 CSA								

* *p* < .05, ** *p* < .01, *** *p* < .001, **** *p* < .0001

^+^ 2 cases were omitted due to missing values

### Regression analysis

**Fig. 2:**
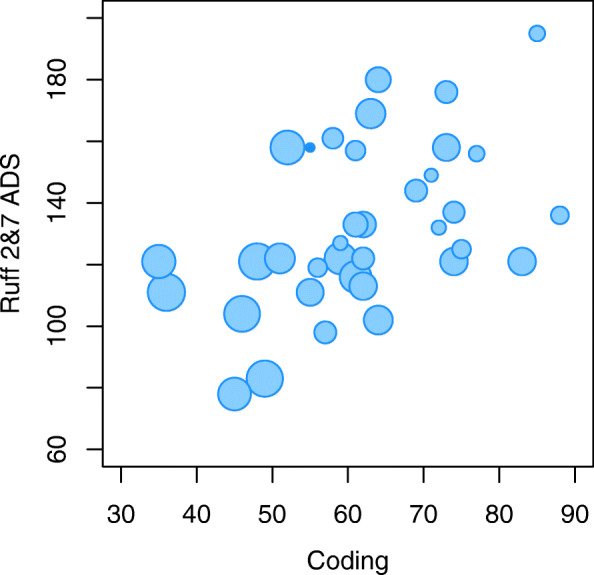
Association between Coding and Ruff 2&7 ADS, with the dots’ size corresponding to MCS’s value, from 0 in the smallest to 33.9% in the largest

A stepwise analysis suggested that two of the predictors of MCS, namely Coding and the Ruff 2&7 ADS contributed to improve a multivariate model, as indicated by a decrease in the model’s Akaike Information Criterion (AIC). In a model including these two predictors, the standardized beta-weights were − 0.514 (*p* < 0.001) and − 0.266 (*p* < 0.1) for Coding and Ruff 2&7 ADS, respectively, and together they accounted for 46.2% (*p* < 0.001) of the total variance in the number of aberrant pauses. The associations are illustrated in Fig. 2, where the dots’ size corresponds to the respondent’s frequency of aberrant pauses. We see that this frequency tends to increase with a decrease in both Ruff 2&7 ADS and Coding. Due to the strong inter-correlations (Table 4), Symbol Search and Ruff 2&7 CSS were discarded from the final model by the stepwise regression analysis.

### Sensitivity and specificity of the MCS

**Fig. 3:**
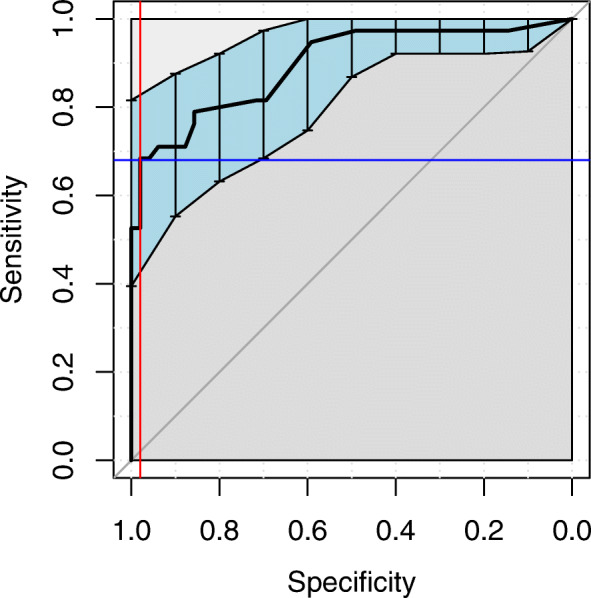
ROC curve for MCS. AUC: 0.896 (0.826–0.965)

The point giving the largest index value was selected as a cut-off [40]. An analysis of the specificity and sensitivity resulted in a prediction AUC: 0.896 (0.826–0.965). Figure 3 indicates that at the cut-off 0.10, the specificity was 0.98 (red line) and sensitivity was 0.68 (blue line).

## Discussion

The results of the present study corroborate SED patients’ subjective complaints, as reflected by their diagnoses, concerning cognitive lapses and fatiguability with a new easily administered tablet-based test, the MCS, in line with earlier results on clinical neuropsychological tests [11, 12]. Thus, the MCS might be regarded as a useful tool that could contribute to diagnosis in a clinical setting.

The MCS test correlated highly with the clinical tests measuring sustained and selective attention, working memory, processing speed, and cognitive control. Performance on Ruff 2&7 ADS and Coding explained almost half of the variance in MCS (46.2%). Although the proportion of SED patients with pathological results on the MCS was very large (78.4%, compared to 3,1% in our normative reference group), performance on the clinical neuropsychological tests was within normal range.

The diagnostic specificity was 0.98 indicating a strong discriminative capacity at the cut-off value (10%) between those who exhibited the specific cognitive dysfunction(s) measured by the MCS and those who did not. This is in line with our previous studies [29, 30, 41]. Sensitivity was, however, lower (0.68), indicating that this type of cognitive dysfunction might occur in some healthy subjects in our normal reference group, but to a much lesser degree than in SED.

The stepwise regression analysis showed that Coding and Automatic detection speed (Ruff 2&7) accounted for 46.2% of the MCS variance, suggesting that processing speed and sustained and selective attention play an essential part in the MCS performance. The explained variance of 46.2% corresponds to a correlation equal to 0.68, which should be characterized as a strong association, for example according to Cohen’s classical guidelines [42].

This means that the number of aberrant pause times is a meaningful indicator of the successful execution of these cognitive control functions and that an escalating number of aberrant pause times might be considered an indicator of impairment of the same cognitive skills required for executing Coding and Ruff 2&7. The high sensitivity of the test and the subsequent regression analysis leads us to believe that it is possible to limit the number of tests for diagnostic purposes for SED patients on an individual level. Variations in earlier studies on group level might be ascribed to differences in tests, regarding the intrinsic quality of the test used in a particular study [43–45] and on the definition of key concepts, such as working memory [44].

Information processing implies dealing with a constant flow of external and internal impulses through a varying number of channels for the different modalities of information. The limited capacity of our brain necessitates the channeling of this information via bottlenecks or gateways [46–48]. The adjustment to different types of stimuli and the choice of an adequate response, i.e., executive tasks, are controlled by selective attention [46] by sharing limited attentional resources through rapid shifts between external and internal modes of attention and working memory [46, 49].

We suggest that the pause times reflect the rapid shifts required to allocate attention within our limited processing capacity [48]. These rapid shifts allow us to alternatively attend to the externally perceived and internally represented environment [27, 50]. Thus, aberrant pause times assessed by the MCS task may reflect a higher switch cost not only between the two domains colour and shape but also a switch cost between internal processes, in this case, short-term memory and external perceptual processes [27].

The higher incidence of aberrant pause times in SED may therefore be indicative of deficits in the capacity for the cognitive switch, resulting in a sluggishness in test performance. Since this cognitive issue occurs in a number of medical conditions indicating impaired information processing capacity it should be regarded as a specific type of cognitive marker rather than a diagnostic marker. We propose that this switch-mechanism, as reflected by pause times in the MCS task, coordinates the management of external and internal impulses and responses. Burgess and colleagues [46] suggest that the rostral part of the prefrontal cortex, Brodmann’s area 10, may play an essential role in this mechanism. However, further neuroimaging studies are warranted to confirm this hypothesis.

### Limitations

The majority of the subjects were women, reflecting the clinical experience that considerably fewer men exhibit SED. Glise and co-workers [51] have investigated possible sex differences in subjects diagnosed with SED and found no behavioural level differences. However, Savic (53) found elevated glutamate levels in the mPFC/ACC and the amygdala in female subjects only. She interpreted the findings of neurobiological differences between the sexes regarding vulnerability to stress. The low frequency of men in the present sample does not ensure the extrapolation of the result to both genders. Further studies are needed concerning possible sex differences in MCS performance. Another limitation is sample size. Although the sample size is quite small, due to large effect sizes the analyses have satisfactory power. For example, power was estimated to be > 0.99 both for the group comparison for the difference between automatic detection and controlled search (62% in our sample compared to 2% among participants in a normative group) as well as for the regression model where Coding and Ruff 2&7 ADS explained 46.2% of the variance in MCS scores. The lack of individual data concerning medication at the time of the testing is a further limitation.

### Clinical applications

Our results indicate that the MCS might replace a larger battery of paper-and pencil clinical tests in the diagnosis of SED. Due to the widespread occurrence of SED, in a busy primary care setting, a comprehensive neuropsychological assessment may be difficult to implement. The test is easily administered on a tablet. It is not sensitive to language, since the primary outcome measure is only the duration of pauses. Results and feedback can be provided immediately after testing, if needed. Since stimuli are presented in random order, the test is repeatable, allowing for the monitoring of progress and treatment effects.

A pedagogical aspect can be included in the feed-back of a patient. Results are presented along with scores also as graphics, giving a visual image of how much information may be lost due to slow processing. The present hypothesis, that the MapCog measures a decrease in the efficiency of the switch mechanisms, might facilitate the understanding of seemingly contradictory clinical phenomena. An example is the observation that patients can perform complex tasks, like a computer task in isolation, but cannot manage seemingly much easier assignments in a complex environment, requiring the interaction with others and the exposure to a larger number of external and internal stimuli.

## Summary and conclusion

The present findings show, for the first time, an abnormal performance in SED by the frequent occurrence of aberrantly prolonged pauses during a serial naming procedure. This finding is in contrast with the normal performance on standard clinical neuropsychological tests of processing speed and working memory. These discrepant test results may be due to the fact that our SED subjects were well educated and may, thus, have had a higher cognitive capacity to perform within the normal range on standard tests, while the pause duration during serial naming was not associated with educational level. The time subjects require to process information during the silent epochs between utterance of coloured shapes may, thus, be a more sensitive indicator of cognitive difficulties in affected subjects. Micropauses between vocal bursts seem to include attentional mechanisms, evidenced by their close association with known attention demanding tests. The present findings demonstrate that the MCS is a measure of attention/and or processing speed, which are ubiqutous prerequisite functions subserving most other cognitive domains. This is especially relevant in the assessment of speeded/time limited versus non-speeded tests. Thus, the absence of correlations with non-time limited tests (e.g., Matrix Reasoning) shows that the MCS helps to disentangle those subcomponents of cognitive abilities.

The present study, therefore, adds new information to the growing evidence of cognitive deficits in SED, which may not be detected otherwise. In addition, another new finding was that participants used different cognitive strategies in their performance of a specific attention demanding test (i.e., RUFF 2&7), suggesting that individual SED subjects managed their performance in different ways to counteract their difficulties. Whether such different strategies also are used by normal subjects, remains to be studied. We conclude that the use of an objective and automatized test measure using a serial naming test paradigm allowed us to disentangle and monitor a specific detail in performance, which we believe is partially associated with deficient selective attentional and attentional switch mechanisms. If confirmed by future studies, this deficiency could be regarded as a cognitive marker shared by different diagnostic groups.
